# Comparative Treatment Outcomes Across Actionable Genomic Alterations in NSCLC: A Large Multicenter Real-World Study

**DOI:** 10.1016/j.jtocrr.2026.101020

**Published:** 2026-05-20

**Authors:** Yasuhiro Kato, Nobuhiko Nishijima, Satoshi Takahashi, Yukari Shirakura, Miwako Omori, Maiko Asai, Aya Fukuizumi, Kakeru Hisakane, Shinji Nakamichi, Susumu Takeuchi, Akihiko Miyanaga, Yoshinobu Saito, Takashi Hirose, Yukio Hosomi, Kazuo Kasahara, Masahiro Seike, Tetsuya Okano, East Japan Chesters Group

**Affiliations:** a Department of Pulmonary Medicine and Oncology, Graduate School of Medicine, Nippon Medical School, Tokyo, Japan; b Department of Pulmonary Medicine and Oncology, Graduate School of Medicine, Nippon Medical School Chiba-Hokusoh Hospital, Chiba, Japan; c Department of Respiratory Medicine, Nippon Medical School Musashikosugi Hospital, Kanagawa, Japan; d Department of Thoracic Surgery, Tokyo Medical University, Tokyo, Japan; e Department of Pulmonary Medicine and Medical Oncology, Nippon Medical School Tamanagayama Hospital, Tokyo, Japan; f Department of Thoracic Oncology and Respiratory Medicine, Tokyo Metropolitan Cancer and Infectious Diseases Center, Komagome Hospital, Tokyo, Japan

**Keywords:** Non–small cell lung cancer, Molecular targeted therapies, Actionable genomic alteration, *EGFR* mutation, Fusion oncogenes, Real-world data

## Abstract

**Introduction:**

Although the efficacy of molecular targeted therapies varies across actionable genomic alterations in NSCLC, comparisons within a single real-world cohort remain limited.

**Methods:**

We conducted a retrospective multi-institutional study of 810 consecutive Japanese patients with advanced or recurrent NSCLC harboring actionable genomic alterations who received molecular targeted therapy from 2017 to 2023. Patients were classified into the following three genomic groups: *EGFR* mutations (group A), *ALK*/*ROS1*/*RET* fusion oncogenes (group B), and others, including *MET* exon 14 skipping, *BRAF V600E*, and *KRAS G12C* mutations (group C). Treatment efficacy and safety were compared across the groups.

**Results:**

Treatment outcomes differed between the subgroups. Group B demonstrated the highest objective response rate (85.8%) and the longest median real-world progression-free survival (rwPFS; 42.5 mo); median overall survival (OS) was not reached. Group A had intermediate outcomes. Group C exhibited the shortest rwPFS (9.2 mo), poorer OS, and higher rates of treatment discontinuation due to adverse events. Such hierarchical differences were observed in patients with baseline central nervous system metastases and in those receiving first-line treatment. In multivariate analysis, genomic subgroup remained associated with rwPFS and OS, with fusion-driven tumors maintaining superior outcomes irrespective of other factors.

**Conclusions:**

This large real-world analysis yielded a hierarchy of therapeutic benefits across actionable genomic alterations in NSCLC. Patients with fusion-driven tumors benefited from targeted therapy, whereas those with *EGFR*-mutated tumors had intermediate outcomes. Treatment of patients with *MET* exon 14 skipping, *BRAF V600E*, and *KRAS G12C* mutations had limited efficacy and higher toxicity, underscoring the need for improved therapeutic strategies.

## Introduction

Lung cancer remains the leading cause of cancer-related mortality worldwide. The prognosis of advanced NSCLC has long been poor, despite improvements in cytotoxic chemotherapy and supportive care.^1^ In recent years, however, the identification of actionable genomic alterations and the development of corresponding molecular targeted therapies have dramatically transformed the therapeutic landscape of NSCLC.^2^, ^3^, ^4^, ^5^, ^6^, ^7^, ^8^, ^9^, ^10^, ^11^, ^12^, ^13^, ^14^, ^15^ Targeted agents are directed at a range of oncogenic drivers, including *EGFR* mutations, *ALK* rearrangements, *ROS1* rearrangements, *RET* fusions, *MET* exon 14 skipping mutations, *BRAF V600E* mutations, and *KRAS G12C* mutations. These have demonstrated clinically meaningful improvements in response rates, real-world progression-free survival (rwPFS), and tolerability compared with conventional chemotherapy in pivotal clinical trials.

Targeted therapies directed at *EGFR* mutations have repeatedly improved outcomes across multiple generations of tyrosine kinase inhibitors (TKIs), culminating in the FLAURA trial, which revealed the superiority of osimertinib in a first-line setting.^2^, ^3^, ^4^ For fusion oncogenes, such as *ALK*, *ROS1* and *RET*, several studies have established the efficacy of TKI.^5^, ^6^, ^7^, ^8^, ^9^, ^10^, ^11^ Similarly, in parallel, agents targeting *MET* exon 14 skipping, *KRAS G12C*, and *BRAF V600E* mutations have demonstrated clinically relevant benefits, although the magnitude of efficacy and the toxicity profile seem to vary by genomic subtype.^12^, ^13^, ^14^, ^15^ Collectively, such data suggest that different molecular-level alterations may exert distinct effects on treatment sensitivity and safety, indicating that actual treatment outcomes are not uniform across genomic subtypes.

Nevertheless, direct comparisons between different actionable genomic alterations remain challenging. Previous clinical trials were conducted independently, with heterogeneous study designs, therapeutic eras, eligibility criteria, and sample sizes. Furthermore, many oncogenic alterations are relatively rare, limiting the feasibility of cross-group comparisons even within real-world data sets. As a result, the clinical and biological differences in outcomes among *EGFR*-mutated tumors, fusion-driven tumors, and tumors harboring other gene alterations have not been comprehensively evaluated within a single, well-defined cohort.

To address this gap, a comparative analysis within a consecutive real-world cohort is essential to delineate the clinical features, treatment efficacy, and toxicity profiles across representative genomic subtypes. In this multicenter retrospective study, we analyzed a large Japanese real-world cohort of patients with advanced NSCLC harboring actionable genomic alterations. Patients were classified into the following three groups: (1) *EGFR* mutation–positive tumors; (2) fusion oncogene–positive tumors (*ALK*, *ROS1*, *RET*); and (3) tumors harboring other oncogenic alterations (*MET*, *KRAS*, *BRAF*, and others). We conducted a comprehensive comparison of clinical characteristics, therapeutic efficacy, and safety profiles among these three groups. We aimed to elucidate differences in real-world outcomes and identify molecular subsets for which future therapeutic development is most urgently needed.

## Methods

### Study Design and Patient Population

This was a multi-institutional, retrospective, observational study conducted across participating centers in Japan. We identified consecutive patients with advanced or recurrent NSCLC who underwent comprehensive genomic testing, were found to harbor actionable genomic alterations, and for whom molecular targeted therapy was initiated between January 2017 and December 2023 across Six sites (Nippon Medical School, Tokyo, Japan; Nippon Medical School Chiba-Hokusoh Hospital, Chiba, Japan; Nippon Medical School Musashikosugi Hospital, Kanagawa, Japan; Tokyo Medical University, Tokyo, Japan; Nippon Medical School Tamanagayama Hospital, Tokyo, Japan; and Tokyo Metropolitan Cancer and Infectious Diseases Center, Komagome Hospital, Tokyo, Japan). Patients were followed until disease progression, death, or the final follow-up date of August 31, 2025, whichever occurred first.

A total of 810 consecutive patients with advanced or recurrent NSCLC who harbored actionable genomic alterations and received molecular targeted therapy were included in the analysis. The study was conducted in accordance with the Declaration of Helsinki and was approved by the institutional review boards of all participating institutions. The requirement for informed consent was waived because of the retrospective nature of the study (Facility Number: 2024-246).

### Genomic Classification

Patients were categorized into three genomic groups according to the detected actionable alterations: group A: *EGFR* mutations, including exon 19 deletions, L858R, and uncommon *EGFR* mutations; group B: Oncogenic fusions involving *ALK*, *ROS1*, or *RET*; and group C: Other actionable alterations, including *MET* exon 14 skipping, *BRAF V600E*, and *KRAS G12C* mutations. Although *NTRK* fusions were prespecified to be included in group B as oncogenic fusion–positive tumors, no patients with an *NTRK* fusion were identified in this cohort. Group C was prespecified as a pragmatic category of non-*EGFR*, nonfusion actionable alterations (*MET* exon 14 skipping, *BRAF V600E*, and *KRAS G12C*). These represent relatively uncommon and clinically heterogeneous subtypes that may be associated with less durable benefits, higher toxicity, or both when treated with currently available targeted agents in real-world practice. This classification was based on current clinical practice and therapeutic indications in Japan during the study period.

### Treatment and Clinical Data Collection

Clinical data were extracted from medical records at each institution and included patient demographics, age, sex, Eastern Cooperative Oncology Group performance status (ECOG PS), smoking history, disease stage at diagnosis, histology, programmed death-ligand 1 (PD-L1) tumor proportion score (TPS), presence of central nervous system (CNS) metastases, treatment line at initiation of targeted therapy, and treatment history, including immune checkpoint inhibitor (ICI). Targeted therapies were administered according to standard clinical practice. Genetic alterations were screened for by an Oncomine Dx target test, Amoy Dx pan lung cancer PCR panel, the Lung Cancer Compact Panel TM, cobas *EGFR* mutation test version (v.2), and immunohistochemical or fluorescence in situ hybridization assays for *ALK* rearrangement. All TPS for PD-L1 were assessed by the 22C3 pharm Dx assay. Information on prior and subsequent ICI therapy was collected and categorized as pretargeted therapy ICI exposure or post-targeted therapy ICI exposure. Imaging assessments were generally performed using computed tomography every 8 to 12 weeks in routine clinical practice; however, the timing and frequency of assessments were not standardized across institutions. Overall response rate (ORR) and disease control rate (DCR) were assessed using Response Evaluation Criteria in Solid Tumors (version 1.1), and the median was used. PFS was assessed as rwPFS, defined as the time from treatment initiation to documented disease progression or death based on physician assessment. Overall survival (OS) was defined as the time from treatment initiation to death from any cause or last follow-up. Adverse events (AEs) were assessed according to Common Terminology Criteria (version 5.0).^16^^,^^17^

### Statistical Analysis

Baseline characteristics were compared across genomic subgroups using Fisher’s exact test for categorical variables. Time-to-event end points, including rwPFS and OS, were estimated using the Kaplan–Meier method and compared using the log-rank test. Cox proportional hazards models were used to estimate hazard ratios (HRs) and 95% confidence intervals (CIs). Multivariable Cox regression analyses were performed to adjust for clinically relevant covariates, including age, sex, ECOG PS, smoking history, disease stage, presence of baseline CNS metastases, treatment line, and history of ICI therapy. The genomic group was included as a key variable of interest in all multivariable models.

Statistical analyses were conducted using EZR (Saitama Medical Centre, Jichi Medical University, Saitama, Japan), a graphical user interface for R that is designed for biostatistics. *p* values less than 0.05 were considered statistically significant.^18^

## Results

### Patient Characteristics

**Figure 1:**
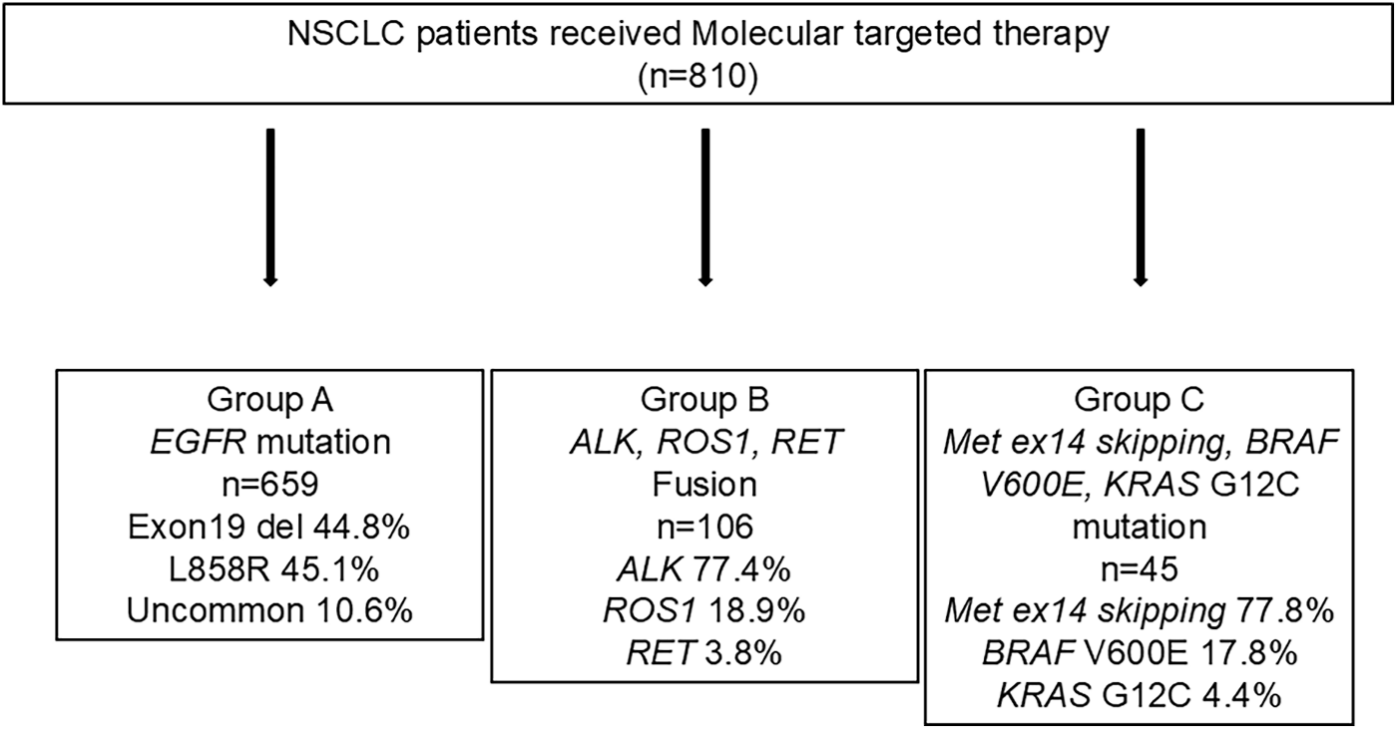
Flow diagram of patient selection and genomic classification. A total of 810 consecutive patients with advanced or recurrent NSCLC harboring actionable genomic alterations who received molecular targeted therapy were included. Patients were classified into the following three genomic groups: group A (*EGFR* mutations), group B (fusion oncogenes including *ALK*, *ROS1*, and *RET*), and group C (other actionable alterations including *MET* exon 14 skipping, *BRAF V600E*, and *KRAS G12C* mutations).

A total of 810 consecutive patients with advanced or recurrent NSCLC harboring actionable genomic alterations who received molecular targeted therapy were included in the analysis. Patients were categorized into the following three genomic groups: group A (*EGFR* mutations; *n* = 659), group B (*ALK*/*ROS1*/*RET* fusions; *n* = 106), and group C (*MET* exon 14 skipping, *BRAF* V600E, or *KRAS G12C* mutations; *n* = 45) (Fig. 1, Table 1).

**Table 1:** Patient Characteristics

Subjects	Group A (*n* = 659)	Group B (*n* = 106)	Group C (*n* = 45)	*p* Value
Age				<0.001^a^
Young (<70 y)	236 (35.8)	70 (66.0)	9 (20.0)	
Elderly (≥70 y)	423 (64.2)	36 (34.0)	36 (80.0)	
Sex				0.011^a^
Men	261 (39.6)	46 (43.4)	17 (37.8)	
Women	398 (60.4)	60 (56.6)	28 (62.2)	
PS				0.33
PS0–1	533 (80.8)	94 (88.7)	38 (84.4)	
PS2–4	114 (17.3)	12 (11.3)	7 (15.6)	
Unknown	12 (1.8)	0 (0.0)	0 (0.0)	
Smoking				0.003^a^
Brinkman index ≥ 100	257 (39.0)	52 (50.9)	27 (60.0)	
Brinkman index < 100	402 (61.0)	54 (49.1)	18 (40.0)	
Stage				0.67
Recurrence	175 (26.6)	32 (30.2)	15 (33.3)	
IIIA–C	25 (3.8)	5 (4.7)	2 (4.4)	
IVA–B	459 (69.7)	69 (65.1)	28 (62.2)	
Histology				<0.001^a^
Ad	649 (98.5)	93 (87.7)	36 (80.0)	
Sq	1 (0.2)	2 (1.9)	4 (8.9)	
Other	9 (1.3)	11 (10.4)	5 (11.1)	
PD-L1				<0.001^a^
≥50%	64 (9.7)	16 (15.1)	11 (24.4)	
1%–49%	163 (24.7)	36 (34.0)	9 (20.0)	
0%	212 (32.1)	37 (34.9)	26 (57.8)	
Unknown	220 (33.4)	17 (16.0)	1 (2.2)	
CNS metastasis				0.12
Yes	211 (32.0)	27 (25.5)	9 (20.0)	
Treatment line				<0.001^a^
1	624 (94.7)	84 (79.2)	35 (77.8)	
2	28 (4.2)	15 (14.2)	10 (22.2)	
≥3	7 (1.1)	7 (6.6)	0 (0.0)	
ICI treatment	94 (14.3)	16 (15.1)	22 (48.9)	<0.001^a^
Post	84 (12.7)	9 (8.5)	15 (33.3)	<0.001^a^
Pre	10 (1.5)	7 (6.6)	7 (15.6)	<0.001^a^

Ad, adenocarcinoma; CNS, central nervous system; ICI, immune checkpoint inhibitor; PD-L1, programmed death-ligand 1; PS, performance status; Sq, squamous cell carcinoma.

a *p* values were calculated using the Fisher’s exact test.

Baseline patient characteristics differed across genomic subgroups (Table 1). Patients in group C were significantly older, with 80.0% aged above or equal to 70 years, compared with 64.2% in group A and 34.0% in group B (*p* < 0.001). A higher proportion of patients in group C had a history of smoking (Brinkman index ≥ 100: 60.0%) than those in group A (39.0%) or group B (50.9%; *p* = 0.003).

Adenocarcinoma was the predominant histology across all groups but was less frequent in group C (80.0%) than in group A (98.5%) or group B (87.7%) (*p* < 0.001). A PD-L1 TPS more than or equal to 50% was more frequently observed in group C (24.4%) than in group A (9.7%) or group B (15.1%) (*p* < 0.001). Baseline CNS metastases tended to be more frequent in group A than in group B or C, although the difference did not reach statistical significance.

Most patients received targeted therapy in a first-line setting; however, later-line treatment was more common in group B or C than in group A (*p* < 0.001). Prior or subsequent ICI treatment was significantly more frequent in group C (48.9%) than in group A (14.3%) or B (15.1%) (*p* < 0.001).

The distribution of molecular targeted therapies differed across genomic groups, reflecting contemporary clinical practice during the study period (Supplementary Fig. 1). In group A, most patients received osimertinib as initial targeted therapy, whereas earlier-generation EGFR inhibitors were administered to a few patients. In group B, next-generation inhibitors targeting *ALK*, *ROS1*, or *RET* were predominantly used. Most patients with *ALK* fusion received alectinib as molecular targeted treatment. In group C, *MET* or *KRAS G12C* inhibitors or *BRAF/MEK* inhibitor combinations constituted the main targeted treatment approaches. Among patients in group C who received *MET* inhibitors, tepotinib was the most frequently administered agent. During the study period, the availability of targeted therapies for certain genomic alterations, including *MET* exon 14 skipping, *BRAF V600E*, and *KRAS G12C*, was limited because of later regulatory approval in Japan.

### Treatment Efficacy

At the data cutoff, the median follow-up time for the entire cohort was 24.9 months. The median follow-up times were 25.6 months in group A, 27.3 months in group B, and 16.1 months in group C. During the follow-up period, a total of 580 progression events and 398 deaths were observed. Progression events occurred in 492 patients in group A, 51 patients in group B, and 37 patients in group C. Deaths were observed in 346 patients in group A, 29 patients in group B, and 23 patients in group C. Treatment efficacy differed markedly across genomic subgroups (Table 2). The ORRs were 74.1% in group A, 85.8% in group B, and 68.9% in group C (*p* = 0.014); the DCRs were 88.1%, 92.4%, and 84.5% in groups A, B, and C, respectively.

**Table 2:** Response of Each Treatment

Response	Group A (*n* = 659)	Group B (*n* = 106)	Group C (*n* = 45)	*p* Value
CR	23 (3.5)	8 (7.5)	1 (2.2)	
PR	465 (70.6)	83 (78.3)	30 (66.7)	
SD	93 (14.1)	8 (7.5)	7 (15.6)	
PD	39 (5.9)	3 (2.8)	3 (6.7)	
NE	39 (5.9)	4 (3.8)	4 (8.9)	
DCR (%)	88.1	92.4	84.5	0.17
ORR (%)	74.1	85.8	68.9	0.014^a^

CR, complete response; DCR, disease control rate; NE, not evaluated; ORR, objective response rate; PD, progressive disease; PR, partial response; SD, stable disease.

a *p* values were calculated using the Fisher’s exact test.

**Figure 2:**
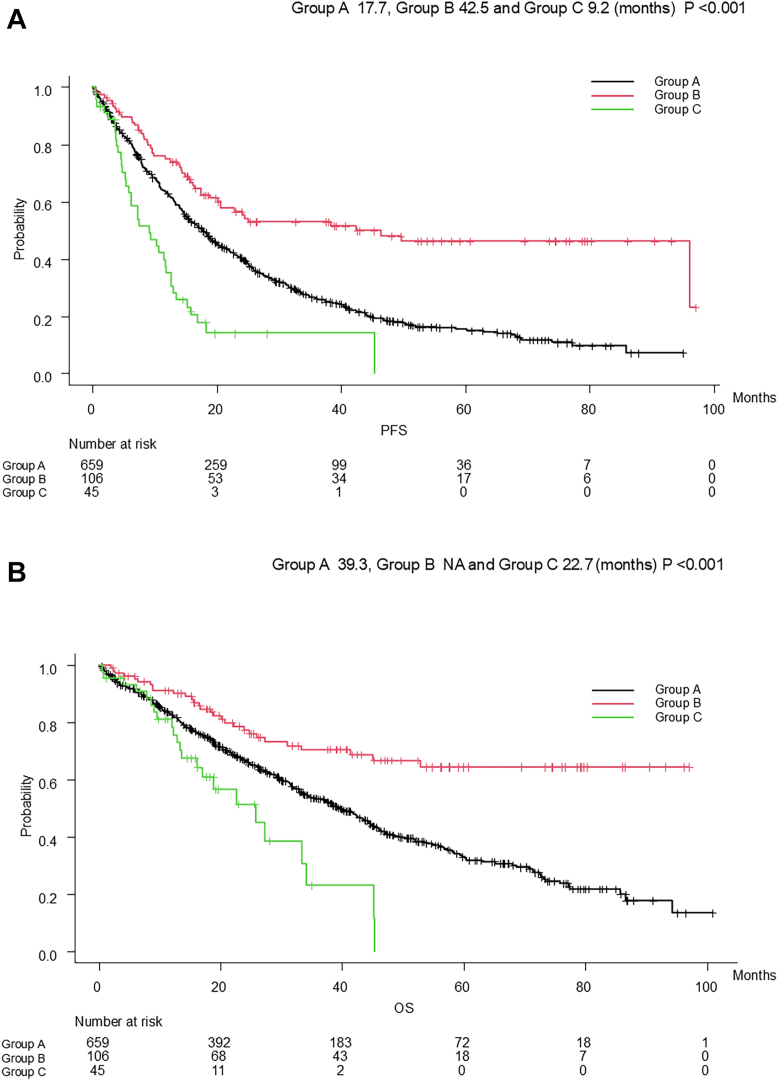
Kaplan–Meier curves for rwPFS (*A*) and OS (*B*) according to genomic subgroup. Patients were stratified into group A (*EGFR* mutations), group B (fusion oncogenes), and group C (other actionable alterations). *p* values were calculated using the log-rank test. Numbers at risk are found below the plots. NA, not reached; OS, overall survival; rwPFS, real-world progression-free survival.

Median rwPFS was 17.5 months (95% CI: 15.9–19.9) in group A, 42.5 months (95% CI: 24.5–not reached) in group B, and 9.2 months (95% CI: 5.4–12.7) in group C (*p* < 0.001). These results indicate a clear gradient in treatment efficacy across genomic subgroups, with group B demonstrating the most prolonged disease control, followed by group A, and the shortest duration being observed in group C (Fig. 2*A*).

Median OS was not reached in group B, 39.3 months (95% CI: 34.7–41.6) in group A, and 22.7 months (95% CI: 16.1–34.2) in group C (*p* < 0.001) (Fig. 2*B*). A similar hierarchical pattern was observed for OS, with the most favorable survival outcome in group B and the least favorable outcome in group C.

Among patients with baseline CNS metastases, a consistent pattern of outcomes was observed across genomic subgroups. The median rwPFS was 20.7 months in group B, 12.6 months in group A, and 11.8 months in group C (Supplementary Fig. 2A). Corresponding OS analyses revealed a similar trend, with group B demonstrating the most favorable survival outcomes (Supplementary Fig. 2B).

In the first-line treatment setting, the hierarchical pattern of efficacy was preserved. Median rwPFS was 96.1 months in group B, 18.5 months in group A, and 9.4 months in group C (*p* < 0.001; Supplementary Fig. 3A). Median OS was not reached in group B, compared with 40.5 months in group A and 25.8 months in group C (*p* < 0.001; Supplementary Fig. 3B). These findings further support the consistent differences in treatment outcomes across genomic subgroups.

To further characterize treatment outcomes, rwPFS and OS were evaluated according to individual genomic alterations (Supplementary Table 1). These exploratory analyses demonstrated that the overall patterns of treatment efficacy were largely consistent with the predefined genomic grouping. Within group A, patients with *EGFR* exon 19 deletion exhibited longer median rwPFS (22.8 mo) and OS (48.7 mo) than those with *L858R* or uncommon *EGFR* mutations. Within group B, patients with *ALK* fusions demonstrated particularly durable responses, with a median rwPFS exceeding 90 months, and median OS was not reached for fusion-positive tumors. Within group C, *MET* exon 14 skipping represented the largest subgroup and was associated with a median rwPFS of 10.4 months, whereas outcomes for *BRAF V600E* and *KRAS G12C* mutations were generally shorter.

Taken together, these findings support the clinical relevance of the genomic classification used in this study, with fusion-positive tumors having the most durable benefit, *EGFR*-mutated tumors demonstrating intermediate outcomes, and tumors harboring *MET* exon 14 skipping, *BRAF V600E*, or *KRAS G12C* mutations exhibiting comparatively limited efficacy.

In univariate analyses, several clinical and molecular factors were significantly associated with rwPFS and OS. Genomic subgroup was a strong determinant of outcome; patients in group B (fusion-driven tumors) demonstrated significantly prolonged rwPFS and OS compared with those in group C, whereas patients in group A had intermediate outcomes (Table 3). Poor PS, presence of baseline CNS metastases, later-line treatment, and prior exposure to ICI were associated with shorter rwPFS. Similar trends were observed for OS, with older age, CNS metastases, and later-line therapy having significant associations with inferior survival outcomes.

**Table 3:** Univariate Analysis and Multivariate Analysis of rwPFS and OS

Analysis	Univariate Analysis			Multivariate Analysis		
rwPFS	HR	95% CI	*p* Value	HR	95% CI	*p* Value
Age (young vs. elderly)	0.87	(0.74–1.03)	0.11	0.95	(0.77–1.16)	0.72
PS (good vs. poor)	0.46	(0.37–0.57)	<0.001	0.52	(0.41–0.66)	<0.001
Sex (male vs. female)	1.28	(1.08–1.51)	0.004	1.12	(0.89–1.38)	0.35
Smoking status (Brinkman index ≥100 vs. <100)	1.38	(1.17–1.62)	<0.001	1.37	(1.10–1.70)	0.005
Treatment line (1 vs. ≥2)	0.69	(0.52–0.92)	0.010	0.63	(0.40–0.98)	0.04
Stage (recurrence or III vs. IV)	0.77	(0.62–0.96)	0.021	0.75	(0.60–0.95)	0.014
CNS metastasis (positive vs. negative)	1.35	(1.14–1.61)	<0.001	1.34	(1.09–1.65)	0.005
Pre ICI (yes vs. no)	1.93	(1.24–2.98)	0.003	1.83	(0.91–3.69)	0.089
Post ICI (yes vs. no)	1.80	(1.45–2.24)	<0.001	1.37	(1.05–1.79)	0.020
Gene mutation (group A vs. group C)	0.47	(0.35–0.63)	<0.001	0.78	(0.51–1.20)	0.25
(group B vs. group C)	0.23	(0.15–0.34)	<0.001	0.30	(0.17–0.50)	<0.001

OS	Univariate Analysis			Multivariate Analysis		
	HR	95% CI	*p* Value	HR	95% CI	*p* Value
Age (young vs. elderly)	0.82	(0.67–1.00)	0.056	1.03	(0.83–1.28)	0.78
PS (good vs. poor)	0.33	(0.26–0.42)	<0.001	0.43	(0.34–0.56)	<0.001
Sex (male vs. female)	1.12	(0.92–1.36)	0.27	1.00	(0.79–1.26)	0.97
Smoking status (Brinkman index ≥ 100 vs. <100)	1.31	(1.07–1.60)	0.008	1.33	(1.04–1.69)	0.022
Treatment line (1 vs. ≥2)	0.78	(0.56–1.11)	0.17	0.72	(0.45–1.15)	0.17
Stage (recurrence or III vs. IV)	0.84	(0.66–1.06)	0.14	0.83	(0.65–1.06)	0.14
CNS metastasis (positive vs. negative)	1.61	(1.30–1.97)	<0.001	1.47	(1.18–1.83)	<0.001
Pre ICI (yes vs. no)	1.54	(0.92–2.59)	0.10	1.79	(0.89–3.62)	0.10
Post ICI (yes vs. no)	1.10	(0.84–1.46)	0.49	1.07	(0.80–1.43)	0.66
Gene mutation (group A vs. group C)	0.56	(0.36–0.85)	0.007	0.78	(0.51–1.20)	0.25
(group B vs. group C)	0.24	(0.15–0.36)	<0.001	0.30	(0.17–0.50)	<0.001

CI, confidence interval; CNS, central nervous system; HR, hazard ratio; ICI, immune checkpoint inhibitor; OS, overall survival; PS, performance status; rwPFS, real-world progression-free survival.

In multivariable Cox regression analysis adjusting for age, ECOG performance status, and line of therapy, genomic subgroup was associated with rwPFS and OS. Compared with group A, group B demonstrated significantly improved outcomes, whereas differences between group A and group C did not consistently reach statistical significance (Table 3).

### Adverse Events

Grade more than or equal to 3 AEs occurred in 24.3% of patients in group A, 19.8% in group B, and 35.6% in group C (*p* = 0.13). Treatment-related interstitial lung disease was observed in 12.1% of group A, 8.5% of group B, and 6.7% of group C patients (Table 4).

**Table 4:** Adverse Event

Event	Group A (*n* = 659)	Group B (*n* = 106)	Group C (*n* = 45)	*p*
AEs > grade3	160 (24.3)	21 (19.8)	16 (35.6)	0.13
ILD	80 (12.1)	9 (8.5)	3 (6.7)	0.4
Treatment-related death	3 (0.5)	1 (0.9)	0 (0.0)	1
Treatment discontinuation reason				<0.001^a^
AE	204 (31.0)	18 (17.0)	16 (36.4)	
Progression	309 (46.9)	36 (34.0)	23 (52.3)	
Ongoing	146 (22.2)	52 (49.1)	5 (11.4)	

AE, adverse event; ILD, interstitial lung disease.

a *p* values were calculated using the Fisher’s exact test.

Treatment discontinuation due to AEs was most frequent in group C (36.4%), compared with group A (31.0%) and group B (17.0%) (*p* < 0.001). Treatment-related deaths were rare across all groups.

## Discussion

This large-scale, multi-institutional study demonstrates profound differences in the clinical behavior of NSCLC cases across distinct actionable genomic alterations. Although differences in the clinical behavior of such alterations have been inferred from previous clinical trials, few studies have comprehensively visualized and directly compared these differences across multiple actionable alterations within a single, large real-world cohort, as was performed in this study.^19^,^20^ A major strength of this study is that, by minimizing confounding from other clinical factors, it highlights the extent to which oncogene addiction and the pharmacologic properties of targeted agents contribute to differences in treatment outcomes across genomic subtypes. Importantly, the observed differences across genomic subtypes remained associated with outcomes after adjustment for clinical factors, suggesting that intrinsic tumor biology, including oncogene addiction, contributes to differential treatment outcomes in clinical practice. These findings suggest that differences in outcomes across genomic subgroups may persist after adjustment for key clinical factors.

The most striking finding of this study is the favorable outcomes observed in fusion-driven tumors (group B), characterized by high response rates, prolonged rwPFS exceeding 40 months, and a median OS that was not reached. These results are consistent with previous clinical trials of next-generation *ALK*, *ROS1*, and *RET* inhibitors, supporting the strong oncogene addiction and durable responses associated with these alterations.^6^, ^7^, ^8^, ^9^, ^10^, ^11^ In addition, accumulating real-world evidence has demonstrated similarly favorable outcomes in fusion-driven NSCLC, and our findings further support these observations in a large, contemporary multicenter cohort.^21^, ^22^, ^23^, ^24^, ^25^, ^26^ Large-scale genomic profiling programs in Europe, such as EPROPA, have highlighted the importance of identifying actionable genomic alterations in clinical practice. In addition, real-world studies from diverse regions, including Latin America, have reported similar trends in survival outcomes, supporting the broader applicability of genomic-driven treatment strategies.^25^^,^^26^ The favorable outcomes in group B were likely influenced, at least in part, by the high efficacy of currently available targeted therapies, particularly ALK inhibitors, which have demonstrated prolonged disease control in both clinical trials and real-world settings. In our cohort, outcomes in group B may have been substantially driven by the ALK-positive population. However, relatively favorable outcomes were also observed in other fusion-positive subgroups (Supplementary Table 1), suggesting that the observed benefit may not be explained solely by treatment efficacy. Rather, shared biological features, such as dependence on fusion-driven oncogenic signaling, may also contribute. Notably, the rwPFS observed in group B in this study seems longer than that reported in pivotal clinical trials such as J-ALEX and CROWN.^5^^,^^27^ Several factors may explain this difference. Real-world cohorts may include patients with more favorable clinical characteristics or indolent disease biology, which can lead to longer observed survival outcomes. In addition, differences in patient selection, treatment patterns, and follow-up duration between clinical trials and routine practice may influence survival estimates. Therefore, these findings should be interpreted in the context of real-world variability. Several factors may explain this difference. Real-world cohorts may include patients with more favorable clinical characteristics or indolent disease biology, which can lead to longer observed survival outcomes. In addition, differences in patient selection, treatment patterns, and follow-up duration between clinical trials and routine practice may influence survival estimates.

By contrast, outcomes for *MET* exon 14 skipping, *BRAF V600E*, and *KRAS G12C* (group C) were consistently inferior across response, rwPFS, OS, and toxicity end points. Although heterogeneity within this group likely contributes to variability, our findings highlight the limitations of currently available targeted therapies for these subtypes and the need for more effective treatment strategies.^12^, ^13^, ^14^, ^15^ Emerging real-world data suggest that, in selected patients with *MET* exon 14 skipping and *BRAF V600E* mutated NSCLC, immunotherapy-based first-line approaches may yield survival outcomes comparable to, and in some cohorts potentially longer than, targeted therapy.^28^, ^29^, ^30^ In parallel, multiple clinical trials are exploring combination strategies incorporating ICIs, cytotoxic chemotherapy, and antibody-based modalities, including antibody–drug conjugates, underscoring the need for continued development of more effective therapies and optimized combinations and sequencing for these molecular subsets.^31^, ^32^, ^33^, ^34^ However, our findings also indicate that molecular targeted therapies for *MET* exon 14 skipping, *BRAF V600E*, and *KRAS G12C* mutations are associated with a relatively high incidence of AEs. This highlights how, in addition to optimizing treatment strategies and sequencing, further improvement in the efficacy and tolerability of targeted agents themselves remains essential for these molecular subsets.

*EGFR*-mutated tumors (group A) demonstrated intermediate outcomes, with robust initial responses and durable disease control, but these did not approach the exceptional activity found in fusion-positive disease. These findings demonstrate a clear hierarchy of therapeutic benefit, with fusion-driven tumors having the most favorable outcomes, followed by *EGFR*-mutated tumors, and with more limited efficacy observed in tumors harboring *MET* exon 14 skipping, *BRAF V600E*, or *KRAS G12C* mutations.

The consistency of this hierarchy across CNS-involved disease and first-line subgroups suggests that intrinsic tumor biology, rather than baseline disease characteristics alone, largely underlies these differences. This emphasizes the clinical importance of accurate genomic classification and highlights areas where therapeutic innovation is urgently needed, particularly in group C.

Several limitations should be acknowledged. First, the retrospective nature of this study introduces the potential for selection bias. Second, treatment selection and sequencing were influenced by drug availability and approval timing during the study period, particularly for alterations included in group C. As a result, *RET* fusions and *KRAS G12C* and *BRAF V600E* mutations were represented by relatively small sample sizes, which may have affected the robustness of subgroup analyses. The relatively small proportion of patients in group C warrants careful consideration. Although differences in the prevalence of oncogenic driver mutations between Asian and Western populations are well recognized, this factor alone does not fully explain the low representation of group C in our cohort. Large-scale genomic screening programs, such as LC-SCRUM, have demonstrated that these alterations occur more frequently than observed in this study. A major contributing factor is likely the timing of regulatory approval and clinical availability of targeted therapies. During the study period, agents targeting *MET exon 14 skipping*, *BRAF V600E*, and *KRAS G12C* were introduced later than those for *EGFR* and *ALK*, which may have limited the inclusion of these patients in routine clinical practice. Therefore, the composition of group C in this study likely reflects a combination of biological prevalence and temporal factors related to drug availability, which should be taken into account when interpreting the results. Treatment outcomes in the *EGFR*-mutated subgroup may have been influenced by temporal changes in the standard of care during the study period from 2017 to 2023, particularly the widespread adoption of first-line osimertinib. This era effect may have contributed to heterogeneity in outcomes within the *EGFR*-mutated group. In addition, uncommon *EGFR* mutations, including de novo *T790M* and exon 20 insertion mutations, were grouped together, which may have influenced treatment selection and observed efficacy. Third, detailed information on co-occurring genomic alterations and the precise timing of ICI exposure was incomplete, potentially confounding clinical outcomes. In addition, detailed information regarding subsequent therapies after recurrence was not systematically available in this data set. Given the retrospective nature of this study, rwPFS was used as the primary end point. As imaging intervals and assessment methods were not standardized, the absolute rwPFS estimates should be interpreted with caution. However, as the study aimed to compare outcomes across genomic subgroups within the same real-world setting, the relative differences observed may still provide clinically meaningful insights. Importantly, consistent hierarchical patterns were observed for both rwPFS and OS, supporting the robustness of our findings despite the inherent limitations of real-world end points. Finally, heterogeneity in treatment regimens and sequencing inherent to real-world clinical practice, including the use of sequential versus upfront next-generation TKIs, could not be fully accounted for.

## Conclusion

In this real-world analysis of 810 patients with NSCLC harboring actionable genomic alterations, fusion-driven tumors demonstrated exceptional clinical outcomes, far surpassing those associated with *EGFR* mutations and other oncogenic drivers. *MET* exon 14, *BRAF V600E*, and *KRAS G12C* mutations were associated with more modest treatment benefits and higher toxicity, identifying clear unmet needs for improved therapeutic approaches. These findings reinforce the importance of genomic subtype in determining prognosis and treatment strategy in NSCLC.

## CRediT Authorship Contribution Statement

**Yasuhiro Kato**: Conceptualization, Study design, Data curation, Methodology, Formal analysis, Investigation, Writing – original draft.

**Nobuhiko Nishijima**: Data curation, Investigation, Writing – review & editing.

**Satoshi Takahashi**: Data curation, Investigation, Writing – review & editing.

**Yukari Shirakura**: Data curation, Investigation, Writing – review & editing.

**Miwako Omori**: Data curation, Investigation, Writing – review & editing.

**Maiko Asai**: Data curation, Investigation, Writing – review & editing.

**Aya Fukuizumi**: Investigation, Study design, Methodology, Writing – review & editing.

**Kakeru Hisakane**: Investigation, Study design, Methodology, Writing – review & editing.

**Shinji Nakamichi**: Investigation, Study design, Methodology, Writing – review & editing.

**Susumu Takeuchi**: Investigation, Study design, Methodology, Writing – review & editing.

**Akihiko Miyanaga**: Investigation, Study design, Methodology, Writing – review & editing.

**Yoshinobu Saito**: Supervision, Writing – review & editing.

**Takashi Hirose**: Supervision, Writing – review & editing.

**Yukio Hosomi**: Supervision, Writing – review & editing.

**Kazuo Kasahara**: Supervision, Writing – review & editing.

**Masahiro Seike**: Supervision, Writing – review & editing.

**Tetsuya Okano**: Formal analysis, Data interpretation, Study design, Methodology, Writing – review & editing.

All authors contributed to the conception and design of the study, critically revised the manuscript for important intellectual content, and approved the final version of the manuscript.

## Disclosure

Dr. Kato has received honoraria from AstraZeneca, Chugai Pharmaceutical, Eli Lilly, and Merck. S. Nakamichi has received honoraria from AstraZeneca, Chugai Pharmaceutical, Takeda, and Amgen. T. Okano has received honoraria from AstraZeneca, Eli Lilly, Pfizer, Chugai Pharmaceutical, Boehringer Ingelheim, and Merck Biopharma. Y. Saito has received honoraria from AstraZeneca, Nippon Boeghringer Ingelheim, and Chugai Pharmaceutical. M. Seike has recived grants or contracts from Chugai Pharmaceutical and Eli Lilly and honoraria from AstraZeneca, Chugai Pharmaceutical, Eli Lilly, Nippon Boehringer Ingelheim, Pfizer, Novartis, and Merck Biopharma. S. Takahashi has received honoraria from AstraZeneca, Chugai Pharmaceutical, Eli Lilly, Takeda, Pfizer, and Amgen. S. Takeuchi has received honoraria from AstraZeneca, Takeda, Eli Lilly, Novartis Japan, and Chugai Pharmaceutical. The remaining authors declare no conflict of interest.
