# Supplementary material for: Comparative Treatment Outcomes Across Actionable Genomic Alterations in NSCLC: A Large Multicenter Real-World Study
Source: JTO Clin Res Rep. 2026 May 20;7(8):101020. doi: 10.1016/j.jtocrr.2026.101020 (PMC13448900; doi:10.1016/j.jtocrr.2026.101020)
Supplement: Supplementary file 1 [file mmc1.pdf]

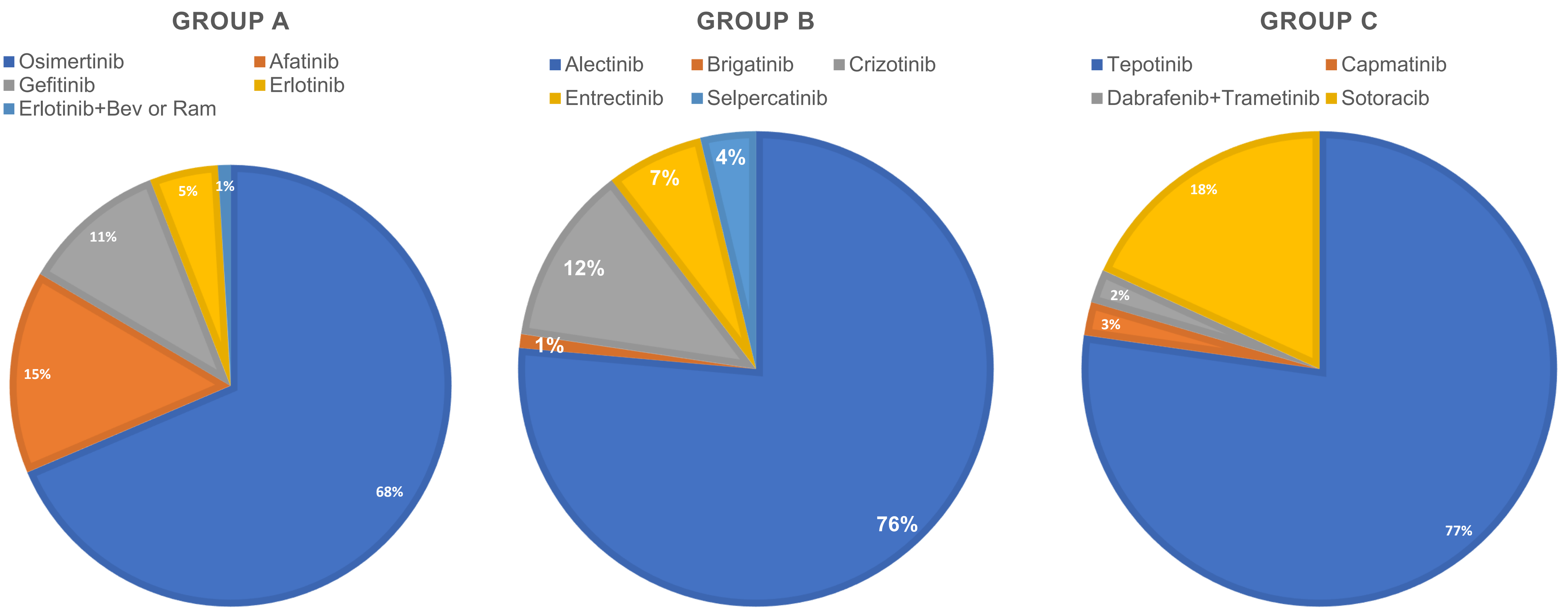
GROUP A
Osimertinib
Afatinib
Gefitinib
Erlotinib
Erlotinib+Bev or Ram
68%
15%
11%
5%
1%
GROUP B
Alectinib
Brigatinib
Crizotinib
Entrectinib
Selpercatinib
76%
1%
12%
7%
4%
GROUP C
Tepotinib
Capmatinib
Dabrafenib+Trametinib
Sotoracib
77%
3%
2%
18%

Suppl Figure 2A

Group A 12.6, Group B 20.7 and Group C 11.8 (months) P <0.001

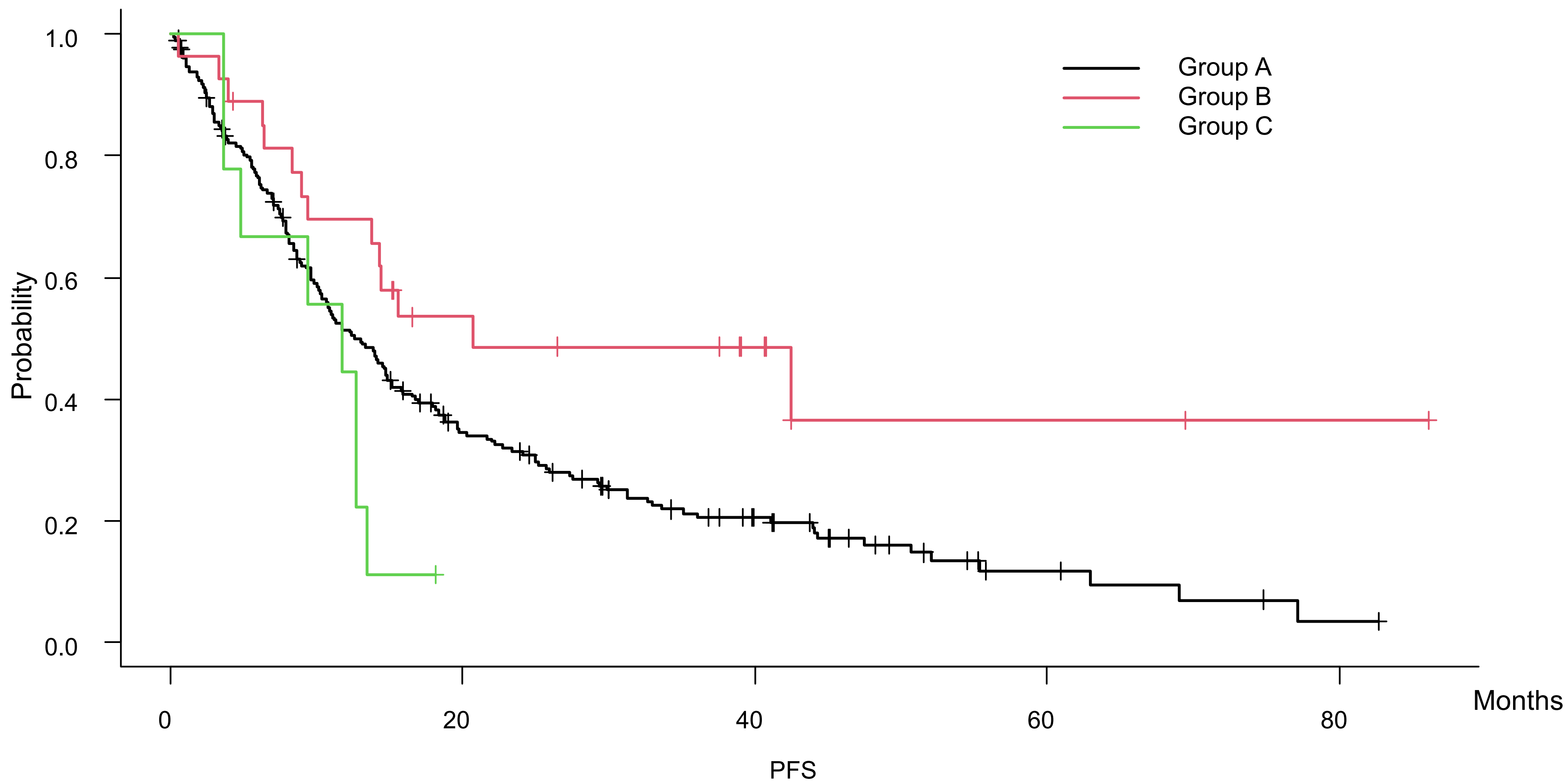

Number at risk

	0	20	40	60	80
Group A	211	64	26	6	1
Group B	27	11	6	2	1
Group C	9	0	0	0	0

Suppl Figure 2B

Group A 27.7, Group B NA and Group C 13.6 (months) P <0.001

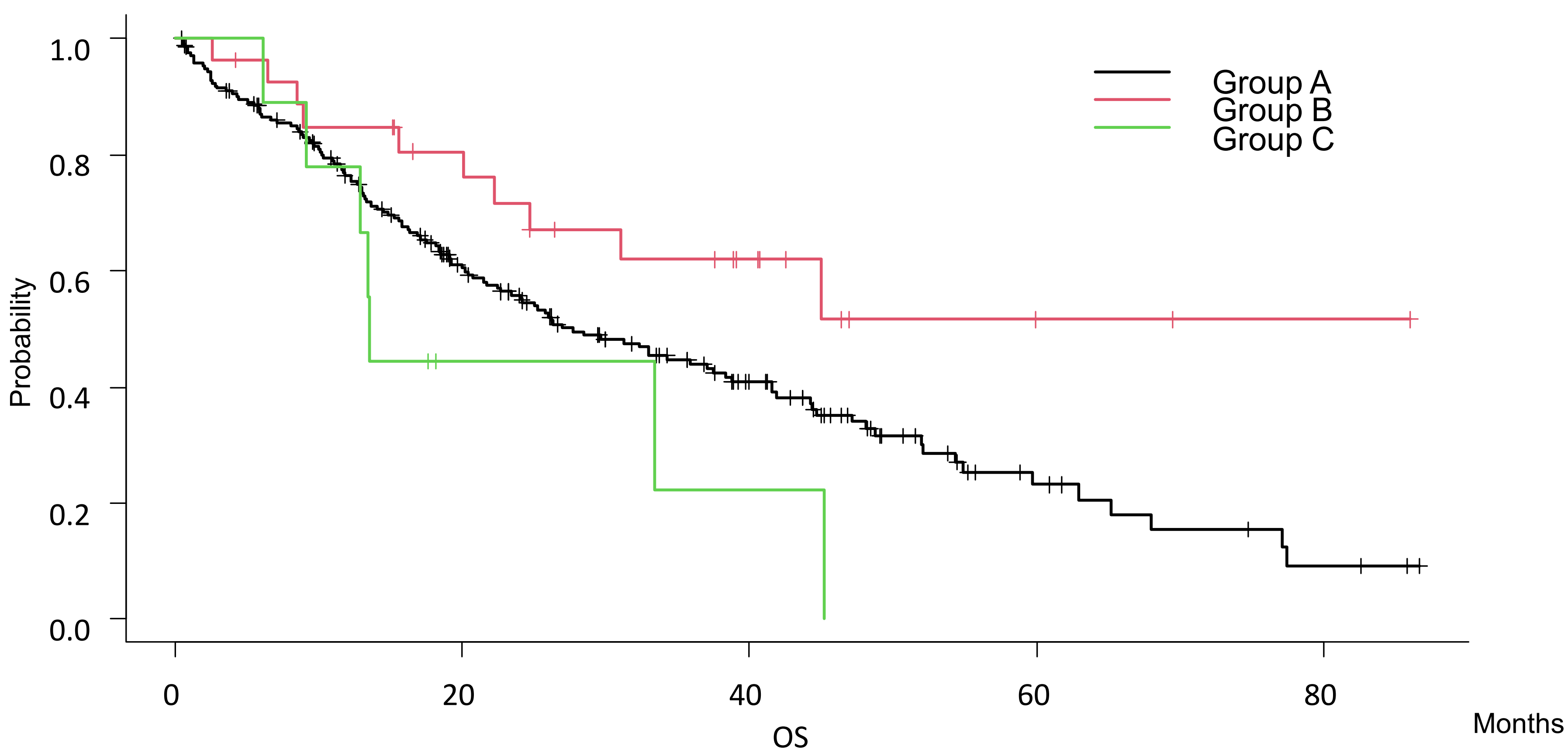

Number at risk

	0	20	40	60	80
Group A	211	106	47	11	3
Group B	27	18	9	2	1
Group C	9	2	1	0	0

Suppl Figure 3A

Group A 18.5, Group B 96.1 and Group C 9.4 (months) P <0.001

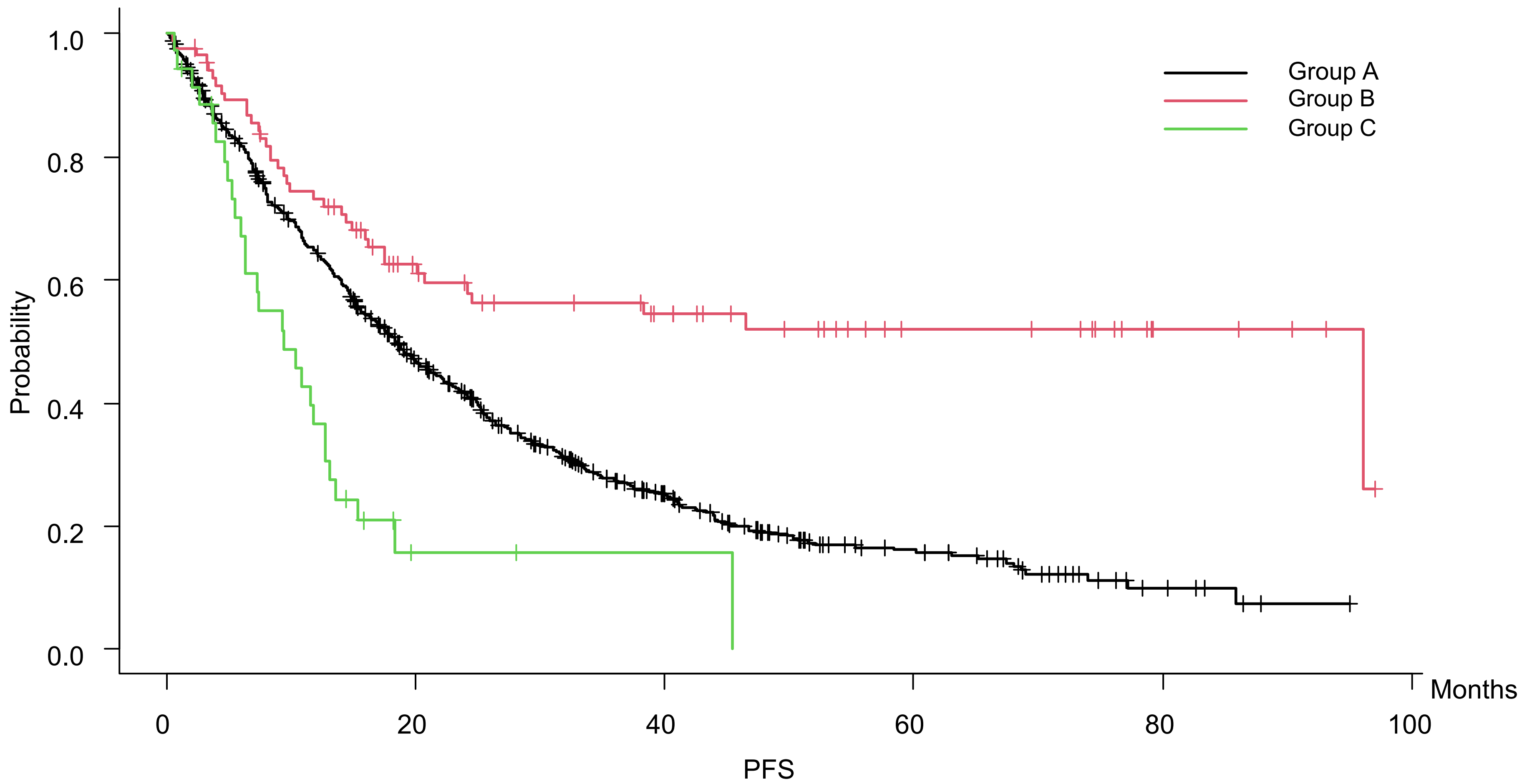

Number at risk

Group A	624	253	98	36	7	0
Group B	84	41	28	14	5	0
Group C	35	2	1	0	0	0

Suppl Figure 3B

Group A 40.1, Group B NA and Group C 25.8 (months) P <0.001

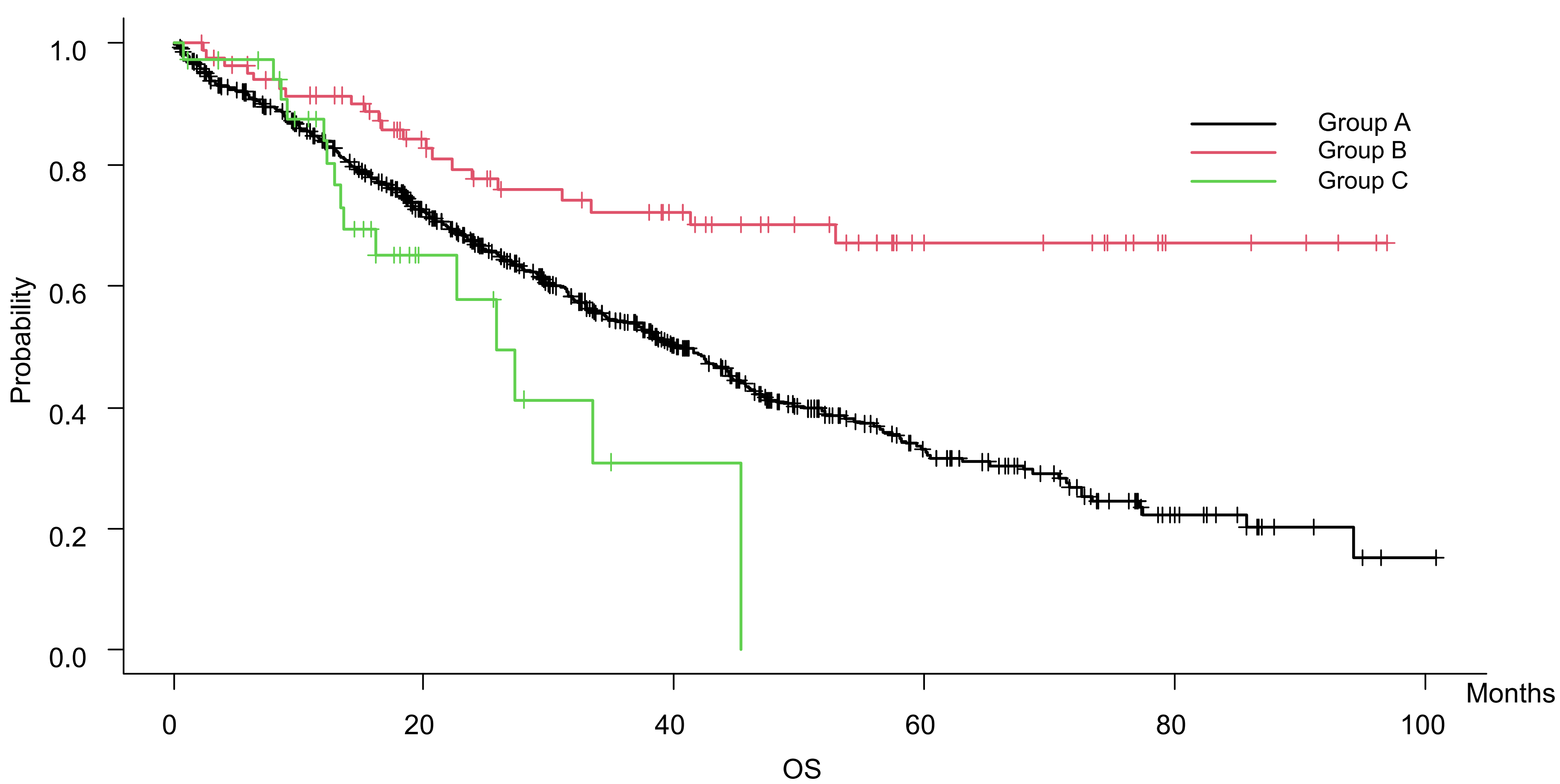

Number at risk

Group A	624	376	176	67	17	1
Group B	84	52	35	14	5	0
Group C	35	9	2	0	0	0
